# Five Cases of Pelvic Disease Treated by Laparotomy
1Read before the Bath and Bristol Branch of the British Medical Association, October 25th, 1899.


**Published:** 1900-12

**Authors:** Ernest W. Hey Groves


### FIVE CASES OF PELVIC DISEASE TREATED BY LAPAROTOMY.[1]

BY

Ernest W. Hey Groves, M.B., B.Sc. (Lond.)

I venture to bring forward these cases as each of them presents special features of interest as regards their diagnosis, treatment, or pathology.

Case I.—**Dermoid Cyst of Ovary.**

K. E., aged 42 years, complained of pain and swelling of legs and painful micturition. She has always been strong and active, never had any serious illness. Catamenia normal. Married 14 years; four

[1] Read before the Bath and Bristol Branch of the British Medical Association, October 25th, 1899.

children; no miscarriages; youngest child four years old; confinements natural. February, 1898, after special exertion during menstruation she complained of a pain in the back and legs. She endured this for two months without advice, till in April, 1898, it became so severe she had to take to her couch. Since August, 1898, she has herself been conscious of a tender mass in the abdomen, and has had great pain and difficulty in micturition. November, 1898, I saw her for the first time, when her condition was as follows: Well nourished and rather stout. Chest normal, with the exception of a soft systolic murmur over the cardiac apex; abdomen rather prominent, resonant all over except in right flank, where a definite tender mass is felt about the size of a fœtal head. Per vaginam and bi-manually uterus in the position of ante-version, rather enlarged, sound passes $3\frac{3}{4}$ inches. The left ovary forms a tender mass in left fornix and in the posterior and right fornices occupying the cavity of the true pelvis is the tumour above referred to, which is however freely movable. The legs show a conspicuous wasting below the knee, the muscles are flabby and there is distinct œdema round both ankles. Pain occurs chiefly in front of both legs and is much worse when the patient stands or walks. Reflexes natural, electrical reaction unaltered. November 30, 1898: Operation. The abdomen was opened by a four-inch incision and a unilocular ovarian cyst of the right side removed without rupture, after ligaturing the pedicle. The left ovary was enlarged and cystic, and was removed. The incision was closed in two layers, peritoneum being closed by a separate silk suture. The wound healed by first intention, recovery was uninterrupted, and the patient returned home on December 19. The tumour removed was a unilocular dermoid ovarian cyst of oval shape, measuring $4\frac{1}{2}$ inches by $3\frac{1}{2}$ inches, containing thick sebaceous fluid and a hard cheesy mass from which several tufts of long hairs were growing. She still cannot walk more than half-a-mile at a stretch, but is able to take an active part in her household duties without pain.

The chief interest of this case lies in the prominence of the pressure symptoms. The muscular wasting was probably due to disuse. If this explanation of the case is correct it is remarkable that such symptoms are not more common, specially in cases of solid pelvic tumours.

Case II.—**Simple Ovarian Cyst causing Menorrhagia.**

P. R., aged 35, unmarried, suffering from severe pelvic pain and menorrhagia. History: has always been anæmic, and has suffered from feeble digestion. About seven years ago her menstrual periods began to be profuse and painful. This condition has gradually increased, so that for the past three years she has been a constant invalid. Menstruation during this time has recurred about every twenty days, and has lasted six to eight days each time, the flow being excessive and accompanied by great pain, especially before and during the first two days of the period. She has lately had constant night sweats, and has felt "feverish." Vomiting and constipation have been frequent. When I saw her first she was thin and extremely anæmic, the lips and conjunctiva being blanched. There was a well-marked hæmic murmur over the pulmonary cardiac area, and she presented the usual symptoms of profound anæmia. On June 2nd, 1900, I examined her under an anæsthetic, and found the uterus anteverted and enlarged, the sound passing $3\frac{1}{2}$ inches. Behind it was a distinctly elastic swelling about the size of a duck's egg. Both the vaginal and rectal mucous membrane moved freely over this. July 1st, 1900. I opened the abdomen expecting to find inflammatory disease of the tubes, or possibly a fibroid tumour, but to my surprise the tumour was the most innocent looking, simple multilocular ovarian cyst of the right side. The left ovary was cystic, and some of the cysts were filled with blood. Both appendages were removed. The patient made an excellent recovery, and although her anæmic condition will make her an invalid for some time, yet she made rapid progress towards good health. A few drops of blood were lost per vaginam on the day following the operation. Besides this she has only once had any subsequent uterine hemorrhage, although slight pelvic sensations of menstruation have recurred at each period.

Both this and the last case are exceptional in the great prominence of pain as a symptom of small uncomplicated ovarian tumours. In both cases, too, there was distinct uterine enlargement. Doubtless the gravity of the symptoms depended on the uterine condition. I should not have removed the left ovary had not the patient's menorrhagia been so marked and the necessity for prompt cure so urgent.

CASE III.—**Uterine Fibroid.**

A. B., aged 44, complains of pains in the abdomen, vaginal bleeding, and general weakness. No previous illness. Married 15 years. No pregnancies. Menstruation regular every 28 days, lasting three days, with little pain, until December, 1898, when her regular period was accompanied with great bearing down pains and profuse hemorrhage. It lasted a week, and after a few days' cessation bleeding re-commenced, and has continued almost daily ever since, a period of two months, in spite of rest and drug treatment. If she remained any time in the erect posture violent flooding resulted. No discharge of matter, no loss of flesh. During the last two months she has had difficulty in defæcation, and greatly increased difficulty in micturition. I first saw her on February 21st, 1899, when her condition was as follows: Patient is fat, weighing $14\frac{1}{2}$ stone. Sallow complexion, and is distinctly anæmic. Nothing abnormal in chest or abdomen. Per vaginam cervix is nulliparous, and is pushed far forwards and to the left. Occupying the posterior and right fornices is a firm elastic swelling, which does not fluctuate. The surface, over which vaginal mucous membrane moves freely, presents gentle irregularities. Sound passes $3\frac{1}{2}$ inches in the concavity backwards and to the right, and gives the impression of not having reached the limit of the uterine cavity. Bi-manually the body of the uterus cannot be felt apart from the pelvic tumour; the latter, which is almost the size of a small child's head, completely fills the pelvic cavity. By firm pressure from the vagina it can be moved slightly, but this causes great pain. Profuse bleeding accompanies vaginal examination. Per rectum, the bowel is normal and its mucous membrane moves freely over the mass in Douglas's pouch. March 1st, 1899, operation. Peritoneal cavity opened by a four-inch incision. Tumour was firmly grasped and lifted out of the pelvis. It was then seen to be a fibroid of the body of the uterus, with a smaller mass growing from the left side, in the region of the cervix. The ovarian arteries were tied and the tumour removed, after passing a temporary ligature round its base. The uterine arteries were separately ligatured on each side of the stump, and anterior and posterior flaps of peritoneum were sewn over the stump. The wound was closed without drainage, and an enema of brandy one ounce and hot water six ounces was given. The patient recovered from the shock with remarkable rapidity, having a pulse of 84 two hours after the operation. March 2nd. No sickness, taking soda and milk. Passed flatus freely by the rectum. Temp. 102°, pulse 124, both steadily rising. March 3rd, second day after operation. Bowels opened three times after turpentine and castor oil enema, taking food well. Temp. 101°, pulse 110. Tongue dry, covered with brown fur. March 7th. General conditions unchanged. No vomiting; bowels moved regularly. Pulse 110, regular, good volume. Abdomen soft. Wound, which healed by first intention, more moist and rather angry-looking. Temp. fluctuates daily between 98° and 103°. Profuse morning sweats. I thought the temperature pointed to some local pelvic suppuration, excluding peritonitis because of her good general condition. She was placed under an anæsthetic, and while moving her a few drops of bloody fluid oozed through the abdominal wound: the latter was therefore opened freely, and dark odourless blood-stained fluid mixed with flakes of lymph escaped. The intestines were matted with lymph, and the deep parts of the wound were ragged and sodden. The abdominal cavity was flushed with several gallons of one per cent. solution of lysol. No attempt was made to remove the lymph from the bowels. A long Keith's tube was placed in Douglas's pouch, and a rubber drain in the upper angle of the wound. The wound was sewn up with fishing gut sutures, a rubber tube being laid in its depth. March 8th. Pulse has not risen above 108 or temp. above 100°. Passes flatus freely. No sickness. Wound dressed every four hours. March 15th. Glass tube shortened, rubber tube removed. Progress good in every respect. Temp. has not risen to 100° since last note. March 18th. Glass tube removed. April 2nd. Whole wound is firmly healed, except at the lowest 1½ inches, where a granulating cavity is left. Patient returned home wearing an abdominal belt. A month later the entire wound was healed, leaving a lineal scar. Patient is now quite well. Can resume her ordinary housework, and walk several miles without fatigue.

Would it have been better in this case to remove the tumour by a vaginal hysterectomy? If we regard merely the size of the tumour such a course would certainly have been possible. You will see that the smaller fibroid mass in the cervical region would have greatly interfered with the ligature of the vessels on that side and have made the vaginal method very difficult and dangerous. But the history of the case after the operation, as bearing upon the details of procedure is the special point to which I wish to draw attention. In many respects the patient's condition was a very remarkable one. Her general condition was so good, she ate and slept well, she never vomited, she had no pain, her bowels were opened regularly, and the pulse was of good volume and only 110. Her hectic temperature was the only danger signal; she was so stout and flabby that the distention was not noticeable, as it would doubtless have been in a thinner subject, and yet the condition could only be called by one name, that of septic peritonitis. But obviously there must be a wide difference between this and the typical case following on operation, when the temperature is often sub-normal, and when a rapid running pulse, vomiting and obstinate constipation, and intense pain are the symptoms which indicate a rapidly approaching fatal termination.

CASE IV.—**Ruptured Ovarian Cyst.**

M. W., aged 43, complains of a sudden attack of violent abdominal pain, accompanied by all the symptoms of collapse. Catamenia normal, regular, last three weeks ago. Married 24 years. Five children, last five years ago. Five years ago she had great pain in left groin, which developed gradually. This continued ten weeks, when it was suddenly relieved by a copious discharge of matter from the vagina. The last two years she has noted a swelling in the lower part of the abdomen, the size of which appeared to fluctuate from time to time. She had abdominal pain of an irregular character all this time. Since Christmas, 1898, swelling has steadily increased, until she was as large as when six months pregnant. March 31st, 1899. After a hard day's spring-cleaning, during which her body felt very heavy and aching, she went to bed about midnight. At 2.0 a.m. she was seized with violent pain in the right part of the lower abdomen. She began to retch. This continued until 11. The patient, who is stout and well-nourished, is very white, with a drawn and anxious expression. Pulse 100, regular, and of good volume. Patient can hardly speak for violent pain and vomiting. These symptoms were relieved by a quarter of a grain of morphia injected sub-cutaneously. The abdomen was much distended, chiefly on the right side, and moves slightly on respiration. The whole of the abdomen below the umbilicus dull to percussion; exquisite tenderness prevents satisfactory examination. The cervix was far forward, not fixed. Posterior fornix and whole of the pelvic floor were bulged down by a tense elastic swelling, which could be felt bi-manually to be continuous with the elastic mass on the right of the abdomen. Diagnosis: cystic tumour, either ruptured or with twisted pedicle. April 2nd, at 9.0 a.m., operation. In opening the peritoneal cavity by the usual incision a quantity of free fluid escaped. It was straw-coloured, and contained several flakes of mucoid lymph. The dark, flaccid ovarian cyst was then found on the right side, about the size of a melon. In handling it a ragged rupture was found on its upper border, which was clamped. The cyst was tapped with a large Wells trocar, and about a quart of greenish fluid drawn off. The cyst appeared to be wholly sub-peritoneal, but showed at one spot, the spot where rupture had taken place, some small secondary cysts. The right Fallopian tube curved over and was lost in its upper border. It was adherent to the sigmoid flexure of the colon by a broad vascular adhesion, which was evidently of old date. The lower part of the cyst descended into the depths of the pelvis. The cyst was removed by a laborious dissection. The abdominal wound was closed in three layers, after a long glass drainage tube had been inserted. The recovery was uninterrupted, and the patient returned home on April 20th, and is now well and strong.

It seems evident that there had been an old tumour in this case, subjected to several inflammatory attacks. Possibly it began as a tubo-ovarian abscess which burst into the vagina five years prior to the operation, and, of course, it may simply be a parovarian cyst.

Case V.—**Tubercular Disease of the Tubes and Right Ovary.**

A. T., 34 years. Complains of pain in the abdomen, and of feeling ill and feverish. Catamenia regular and normal. Married 11 years. No pregnancies. For the last nine years she has suffered from a bearing down pain, chiefly on the left side, but has never been confined to bed. Has worn a variety of pessaries and electric belts without benefit. Last November the pain became much worse, and has increased until now, March 31st. She is bed-ridden, the pain being so great that she cannot put her foot to the ground. On this date her condition was as follows: temperature 102.5°, pulse 124, abdomen rigid, the lower part being distended and immovable. A very tender mass could be felt in Douglas's pouch, and in the left posterior quarter of the pelvis. This was fixed to the uterus, and could not be differentiated from it. The condition evidently being one of acute pelvic peritonitis, she was admitted to my Home, and for ten days shewed some improvement; then the temperature began to show evening rises, and on May 13th I have the following note: During the past four weeks the patient has steadily lost ground. The temperature never remained normal during 24 hours, making an evening rise to 101 or 103. Profuse sweats occurred at night and in the early morning. Appetite has been very bad, and there is often slight vomiting. Emaciation has been marked. Locally the general fixation and tenderness have diminished, but otherwise the condition is unchanged. No soft spots can be felt, either by vagina or rectum. May 17th. Peritoneal cavity opened with some difficulty owing to the fact that the omentum was adherent to the abdominal wall and to the pelvic contents. It had to be tied and cut in places. Where the pelvic contents were exposed coils of small intestine were seen adherent to one another and to the uterus and its appendages; they were covered with gray miliary tubercle. The bladder was drawn up and adherent to the abdominal wall, and would have been cut into but for the guidance of a sound. The left Fallopian tube was dilated, forming a pyo-salpynx, about the size of a hen's egg. It was closely adherent to the sigmoid flexure of the colon, and to a coil of small intestine. After it had been removed the right appendages came into view. They formed a soft friable suppurating mass, closely adherent to the uterus in front and the rectum behind. The mass was enucleated with extreme difficulty, the right ureter being exposed and narrowly escaping ligature. The left ovary appeared to be healthy, and was therefore not removed. Abdomen was irrigated with salt solution and sewn up, an iodoform gauze drain being left. A quart of hot salt solution with one ounce of brandy was administered as an enema, whilst on the table. May 19th. Patient had recovered from shock well. The wound had been dressed every four hours. To-day the discharge had a fæcal odour. May 20th. Bowels opened after an enema. There was a copious discharge of fluid, fæcal matter and gas from the wound; the fistula was therefore probably connected with the small intestine. May 26th. Condition has become worse. The whole of the wound has been opened up. Temperature rises to 101° in the evening; pulse always over 120. Feeling the condition was desperate, she was again put under an anæsthetic and the pelvic cavity well washed out. The perforation in the bowel was found with difficulty; it was in the coil of small bowel which had been adherent to the pyo-salpynx. The bowel was so densely fixed by adhesions that it could not be brought to the surface, and the tension put on stitches in order to bring the edges of the opening together, broke through the friable tissue. The attempt to mend the rent was abandoned, a rubber drain passed from Douglas's pouch into the vagina, and a gauze drain left in the abdominal wound. The patient died about twenty-four hours after, eleven days after the original operation.

The clinical features of this case were those of an acute septic disease, shewing no tendency to yield to expectant treatment. Practically the patient was confined to bed six weeks; nevertheless the hectic fever increased, and in every way she went from bad to worse, so that in spite of the great danger of the operation, and in face of the unfortunate result in this case, it seems to me that surgical treatment offered the only chance of life.

